# Removal of alachlor from water by catalyzed ozonation on Cu/Al_2_O_3_ honeycomb

**DOI:** 10.1186/1752-153X-7-143

**Published:** 2013-08-27

**Authors:** Haiyan Li, Yan Huang, Shuang Cui

**Affiliations:** 1 Key Laboratory of Urban Stormwater System and Water Environment (Beijing University of Civil Engineering and Architecture), Ministry of Education, Beijing 100044, China; 2 Beijing Municipal Environmental Monitoring Centre, Beijing, 100048, China

**Keywords:** Catalytic ozonation, Alachlor, Degradation

## Abstract

**Background:**

The herbicide alachlor (2-chloro-2′6′-diethyl-N-methoxymethylacetanilide) has been known as a probable human carcinogen, and the MCL (minimum contamination level) for drinking water has been set at 2 μg L^-1^. Therefore, the advanced methods for effectively removing it from water are a matter of interest. Catalyzed ozonation is a promising method for refractory organics degradation. Cu/Al_2_O_3_ catalyzed ozonation for degrading an endocrine disruptor (alachlor) in water was investigated.

**Results:**

Experimental results showed that the ozonation of alachlor can be effectively catalyzed and enhanced by Cu/Al_2_O_3_-honeycomb. The main intermediate products formed (aliphatic carboxylic acids) were mineralized to a large extent in the catalytic process.

**Conclusions:**

This study has shown that Cu/Al_2_O_3_-honeycomb is a feasible and efficient catalyst in the ozonation of alachlor in water. Less intermediate oxidation product was produced in the catalytic process than in the uncatalytic one. Furthermore, the mineralization of alachlor could be enhanced by increasing the pH of the reaction solution.

## Background

The herbicide alachlor (2-chloro-2′6′-diethyl-N-methoxymethyl acetanilide) has been found in ground water and surface water in many parts of North America [1,2]. Alachlor is a probable human carcinogen, and the MCL (minimum contamination level) for drinking water has been set at 2 μg L^-1^[3]. Therefore, the advanced methods for effectively removing it from water are a matter of interest.

Beltran has found that ozone can degrade alachlor in drinking water effectively, but a very low TOC (Total Organic Carbon) removal percentage was obtained [4]. This fact has led to further research on how to enhance the efficiency of ozonation, and the catalytic ozonation process was developed [5,6].

It has been shown that metal-catalyzed ozonation can destroy hazardous materials and increase the removal efficiency of organic compounds such as oxalic acid, formic acid and humic substances in water [7-16]. It was found that Fe^2+^ and Mn^2+^ could catalyze the ozonation of alachlor in water [17]. These experimental results demonstrated that metal-catalyzed ozonation has the potential to oxidize the refractory pollutants and degrade the oxidized intermediate products. However, the biggest disadvantage of metal-catalyzed ozonation is the difficulty of the separation of the solid–liquid phase. Therefore, the development of supported metal catalysts in ozonation, which can avoid the problem of separation, has drawn great attention [18].

The aim of our activity was to experimentally investigate the mineralization of alachlor in heterogeneous catalyzed ozonation by applying Cu/Al_2_O_3_.

### Experimental

Alachlor (99% purity) was obtained from AccuStandard Inc. (USA). Synthetic raw stock alachlor solution was prepared by dissolving 200 mg alachlor into 1000 mL ultra-pure water (18 MΩ) to afford a solution of 200 mg L^-1^, and the pH level of the solution was 6.39. Solutions of 100 mg L^-1^ and 10 mg L^-1^ alachlor for tests were obtained by diluting the above stock raw solution.

The supported catalysts of Cu/Al_2_O_3_ were prepared by an impregnation method with an aqueous solution of copper nitrate, followed by evaporation for drying in a rotary evaporator under pressure reduction at 333 K. The wet sample was dried at 393 K for 12 h and then calcined at 873 K for 3 h in air.

Washcoated honeycomb catalysts were prepared using Cu/Al_2_O_3_ powders. Mixing 100 g powder and 400 g water using a ball mill prepared wash-coat slurries. Cordierite honeycombs with 400 cells per square inch were dipped into the washcoat slips and excess slurry was blown out with an air knife. The samples were then dried and calcinated at 873 K for 3 h. The washcoat loading was 110 g L^-1^ after calcinations.

We carried out three experiments: the degradation of alachlor in the uncatalyzed ozonation process, the catalyzed ozonation of alachlor, variation of pH in alachlor ozonation process and the effect of pH on ozonation.

## Methods

BET surface area, pore volume and pore diameter were obtained from N2 adsorption isotherms measured at 77 K using an ASAP 2000 instrument (Mi-cromeritics Co., USA). The catalysts were characterized by X-ray diffractometry using a computerized Rigaku D/max-RB X-Ray Diffractometer (Japan, Cu Kα radiation, 1.54056 nm).

### Ozonation methods

Ozone was generated from oxygen by a laboratory ozonizer (Mitsubishi, ozonizer series OS-1N, Japan). The gaseous concentration of ozone was 12.2 mg L^-1^ min^-1^, and the flow rate was controlled at 40 mL min^-1^. The experiments were performed in a stainless reactor (200 mm high, 30 mm i.d.), which was equipped with a Ti porous plate (10 μm porous size) at its bottom to distribute the dosed ozone and obtain smaller gas bubbles. The Cu/Al_2_O_3_ catalyst powder was dispersed into the reaction solution when the Cu/Al_2_O_3_ powder was applied. In the Cu/Al_2_O_3_-honeycomb catalyzed ozonation, the honeycomb cordierite coated with Cu/Al_2_O_3_ was placed on the porous plate. In uncatalyzed ozonation, a honeycomb cordierite without a Cu/Al_2_O_3_ coating but of the same size and bulk as that used in the process with catalytic ozonation was placed in the reactor. Ozone gas flowed upward through the holes in the cordierite to contact sufficiently with the coated catalyst when the stationary state regime was attained. To absorb the excess ozone, effluent gas was introduced into a glass bottle containing 300 mL of 2% KI solution. During the reaction, 4 mL of treated solution was taken out at regular intervals, followed by the introduction of nitrogen into the sample bottle to blow out the residual ozone to stop the reaction. 2 mL of the sample was used to analyze the concentrations of alachlor and such intermediate products as organic acids directly. The other 2 mL was used to determine TOC after the samples had been diluted for 7.5 factors.

### Chemical analysis

The gaseous ozone concentration was iodometrically measured according to the iodometric method for the determination of ozone [19]. The concentration of residual alachlor was measured directly using HPLC (Shimadzu, 10A) with a UV-detector after ozonation. Pure ACN (acctonitrile)(99.8%) and HPLC grade water with 50 mmol/L ammonium acetate were used as mobile phase. The detection length for the analysis of alachlor in the HPLC analysis was 20.468 min [20]. Aliphatic carboxylic acids produced during the ozonation period were analyzed using an ion chromatograph (Dix-500, Dionex Co.) equipped with a conductivity detector and an AG9-HC guard column (Dionex Co.). We identified the acids with ion chromatograph by the comparision between real peaks and the standards. The eluent was 1.8 mM Na_2_CO_3_ and 1.7 mM NaHCO_3_ solution.

For the determination of hydroxyl radicals generated in the ozonation process, EPR experiment was applied. A nitron spin-trapping reagent DMPO was used in the process. The powdered Cu/Al_2_O_3_ was selected for EPR experiments. Alachlor stock solution, O_3_ and the catalyst powder were mixed with DMPO in ultra-pure water (18 MΩ). Immediately after the mixing, 25 L of the solution was transferred into a capillary tube, and EPR spectra were recoded in the X-band on a Bruker ESP spectrometer at room temperature. EPR measurements were conducted under the following conditions: modulation amplitude 2.0 G; microwave power, 10.00 mW; modulation frequency 100 Khz, sweep width 100.0 G, and receiver gain 1.00e+005.

## Results and discussion

**Figure 1:**
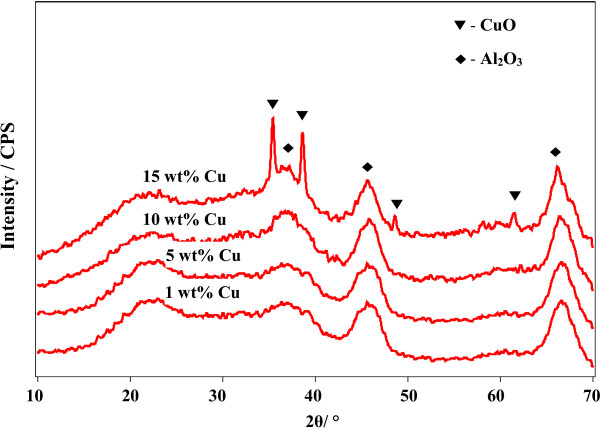
**XRD patterns of Cu-Al**_**2**_**O**_**3 **_**catalysts with different Cu loadings.**

The characteristics of the catalysts with different Cu loadings are shown in Table 1 and Figure 1. The Al_2_O_3_ (gamma type) used in the catalysts had the most favorable performance, with a Cu loading of 10 wt.% and a total BET surface area of 170 m^2^ g^-1^. Thus the prepared Cu/Al_2_O_3_ catalyst powder was mounted on a square-celled extruded cordierite in a honeycomb shape (400 cell inch^-2^, 100 mm high, 28 mm i.d).

**Table 1:** **BET surface area, pore volume and pore diameter of Cu-Al**_**2**_**O**_**3 **_**catalysts with different Cu loadings**

**Catalyst**	**BET surface area(m**^**2 **^**g**^**-1**^**)**	**Pore volume(cc g**^**-1**^**)**	**Pore diameter(A)**
1 wt.% Cu-Al_2_O_3_	235.1	0.7214	122.8
5 wt.% Cu-Al_2_O_3_	205.1	0.6280	122.58
10 wt.% Cu-Al_2_O_3_	171.9	0.5298	123.3
15 wt.% Cu-Al_2_O_3_	147.7	0.4839	128.5

### The degradation of alachlor in the uncatalyzed ozonation process

**Figure 2:**
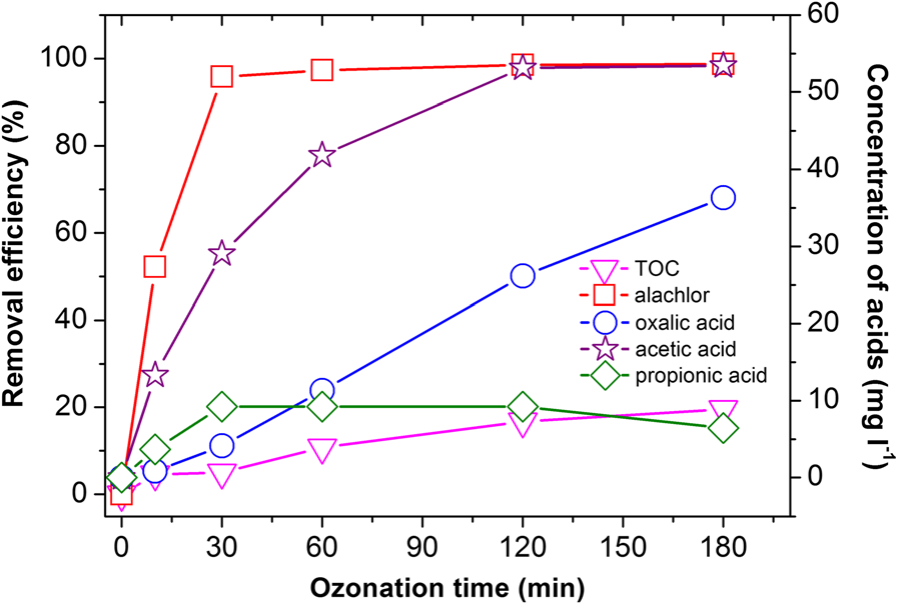
**Degradation of alachlor when ozone was applied alone and acids produced in the reaction process: (**□**) alachlor; (**▽**) TOC; (**☆) acetic acid; (○) oxalic acid; (◇**) propionic acid.** T: 20°C, Alachlor concentration: 100 mg L^-1^.

The total organic carbon (TOC) removal rate was chosen as an overall kinetic parameter to evaluate the mineralization efficiency of the alachlor solution. As shown in Figure 2, alachlor could be effectively degraded with a percentage of above 95% within 30 min of ozonation when ozone was applied alone. However, a modest decrease in TOC of about 20% was achieved after an ozonation period of 180 min. This phenomenon demonstrated that in the reacting condition of our study, alachlor could not be degraded to CO_2_ completely by ozone alone. Intermediate oxidation products which could not be degraded easily by ozone were produced. They included mainly short chain mono- and di-carboxylic acids such as acetic acid, propionic acid and oxalic acid (Figure 2). When the solution was ozonated for 180 min, the concentrations of acetic acid, propionic acid and oxalic acid reached 53.4 mg L^-1^, 6.45 mg L^-1^ and 36.3 mg L^-1^ respectively, and no further decreasing trend was observed. The reason for the accumulation of acids in the solution that the activity of low molecular fatty acids with ozone and •OH are both very low except for formic acid [21], and much great number of •OH is needed to oxidize these carboxylic acids. However, in the ozonation of alachlor by ozone alone, there was not enough •OH produced from the decomposition of ozone to degrade these acids. Therefore, the decomposition of these acids was considered to be the rate-determining step of alachlor mineralization in the ozonation process.

### The catalyzed ozonation of alachlor

**Figure 3:**
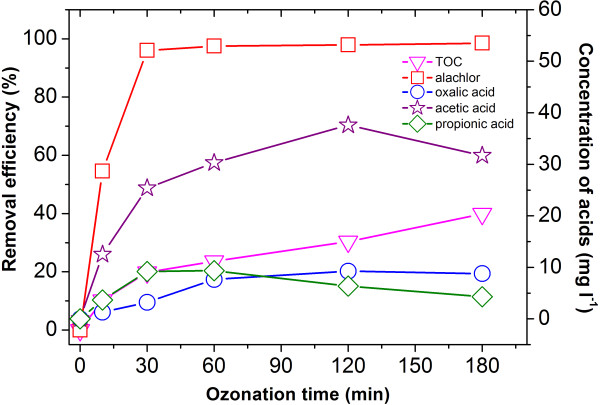
**Degradation of alachlor with catalytic ozonation by Cu/Al**_**2**_**O**_**3 **_**powder and the corresponding acids produced: (**□**) alachlor; (**▽**) TOC; (**☆**) acetic acid; (○) oxalic acid; (**◇**)propionic acid.** T: 20°C, Alachlor concentration: 100 mg L^-1^, the dosage of Cu/Al_2_O_3_ powder: 0.27g L^-1^.

The degradation of alachlor in the Cu/Al_2_O_3_ powder catalyzed ozonation process is shown in Figure 3. Nearly the same alachlor degradation percentage was obtained for samples with or without the catalyst applied. However, the TOC removal rate was raised about 20%, and less aliphatic carboxylic acids were produced in the ozonation process with catalyzation. The experimental results above showed that the catalytic effect was presented when Cu/Al_2_O_3_ powder was applied. More •OH was produced in the process with catalyzation than in the process without catalyzation, which could be verified by the comparison of EPR results (the DMPO-OH signal in the catalyzed and uncatalyzed ozonation processes were 10000 and 2500 respectively) [19]. Large amount of •OH produced in the process with catalyzation was capable of achieving the removal of the acids. The results above verified that applying Cu/Al_2_O_3_ to the ozonation of alachlor was feasible and efficient.

To modify the ability of the catalyst effectively and produce more •OH for catalyzing the ozonation of alachlor, Cu/Al_2_O_3_-honeycomb catalyzed ozonation was studied.

**Figure 4:**
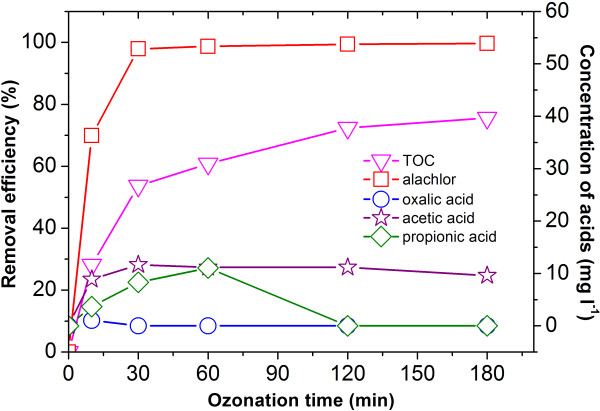
**Degradation of alachlor with catalytic ozonation by Cu/Al**_**2**_**O**_**3**_**-honeycomb and the corresponding acids produced: (**□**) alachlor; (**▽**) TOC; (**☆**) acetic acid; (**○) oxalic acid; (◇)propionic acid. T:20°C, Alachlor concentration: 100 mg L^-1^.

As shown in Figure 4, Cu/Al_2_O_3_-honeycomb catalyzed ozonation led to 75.3% of TOC removal from water after 180 min of ozonation, which was much higher than that obtained in the ozonation using dispersed Cu/Al_2_O_3_ powder. The production of aliphatic carboxylic acids could explain this phenomenon very well (Figure 4). In the Cu/Al_2_O_3_-honeycomb catalyzed ozonation, the maximum concentrations of produced acetic acid, propionic acid and oxalic acid were 11.7 mg L^-1^, 11.0 mg L^-1^, and 1.03 mg L^-1^ respectively, which were lower than those in the Cu/Al_2_O_3_ powder catalytic process. Furthermore, the concentrations of propionic acid and oxalic acid were below the detection limit of the method and instrumentation used after 120 min of catalytic ozonation. It could be concluded that coated Cu/Al_2_O_3_-honeycomb catalyst showed higher catalytic efficiency than the powdered catalyst in the ozonation process of alachlor. The proposed heterogeneous catalytic ozonation mechanism could be expressed as follows: (i) simultaneous adsorption of ozone and alachlor molecules on the catalyst surface, (ii) decomposition of ozone on the metallic sites and production of surface bound hydroxyl radicals that is more reactive than ozone, (iii) oxidation of adsorbed alachlor molecules by adjacent hydroxyl radicals. Oxidation proceeds through several oxidized intermediates whilst hydroxyl radicals are continuously generated by dissolved ozone that is transferred to the catalyst surface. The affinity of the oxidation products to the catalyst decreases and final oxidation products desorb from the catalyst surface [22]. More •OH was produced in this process than in the Cu/Al_2_O_3_ powder catalyzed ozonation process (the DMPO-OH signal increased from 10000 to 45000), and the catalytic reaction would be subsequently more sufficient. As a result, alachlor was mineralized to a larger extent.

### Variation of pH in alachlor ozonation process and the effect of pH on ozonation

#### Variation of pH in ozonation

**Figure 5:**
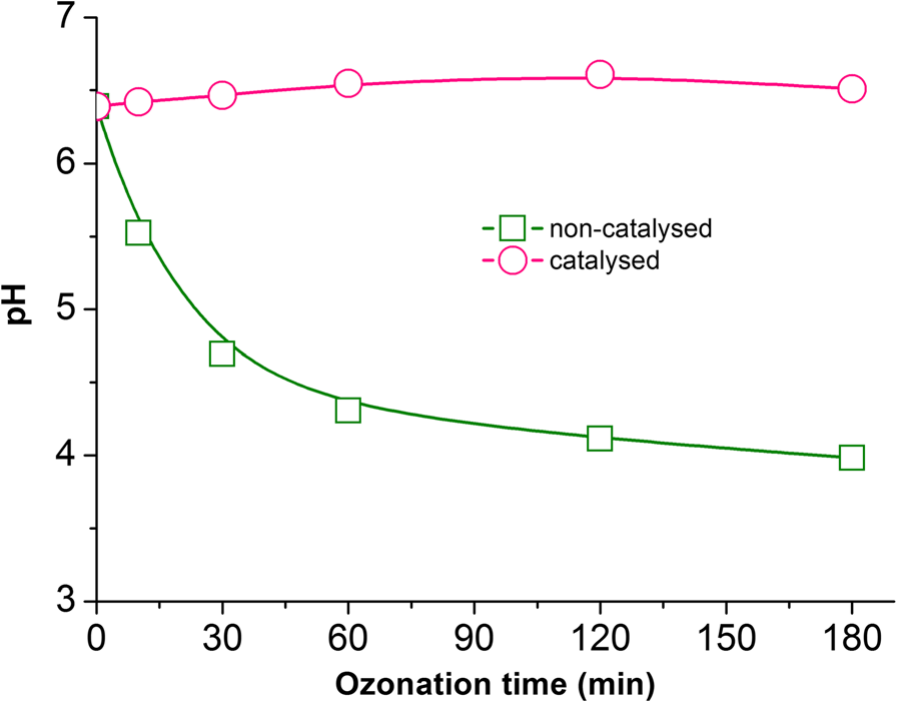
**The shift in pH as a function of time before and after ozonation: (**□**) uncatalyzed process; (**○) catalyzed process. T: 20°C, Alachlor concentration: 100 mg L^-1^.

As shown in Figure 2, a large amount of organic acids was produced in the uncatalyzed ozonation, which led to an obvious pH shift from 6.39 to 3.98 after 180 min of ozonation as shown in Figure 5. The mineralization rate of alachlor by ozone was correspondingly affected by the decrease of pH. Organic hazardous substances react with ozone following two paths: a direct reaction with molecular ozone and an indirect reaction with the radical species formed when ozone decomposes in water. At a higher pH, •OH is inspired, and the main reacting route follows a radical pathway, but at a lower pH, it follows a selective direct reaction pathway. Since the oxidation potential of hydroxyl radicals (2.80) is much higher than that of molecular ozone (2.07), a stronger ability to oxidize and a more rapid reaction are presented in a radical reaction than in a direct reaction. Therefore, with the decreasing of the alachlor solution’s pH in the uncatalyzed ozonation, the direct reaction between alachlor and ozone played a dominant role and the ability to oxidize became weaker, which was unfavorable to the mineralization of alachlor.

However, the pH of the Cu/Al_2_O_3_-honeycomb catalyzed solution remained nearly stable at a level of 6.15-6.45 throughout the ozonation process. This phenomenon could be due to the fact that smaller amount of each type of aliphatic carboxylic acid formed during the catalytic ozonation process than during the uncatalytic ozonation process. The amount of these acids was not enough to cause the pH of the reaction solution to decrease. Since a higher pH level was obtained in Cu/Al_2_O_3_-honeycomb catalyzed ozonation than in the uncatalyzed one, the radical reaction played the dominant role and the process with catalyzation demonstrated more efficient oxidization. Thereafter, most of the acids produced in the ozonation were removed and greater mineralization of alachlor was achieved.

#### The effect of the initial pH of the reaction solution on the alachlor ozonation

**Figure 6:**
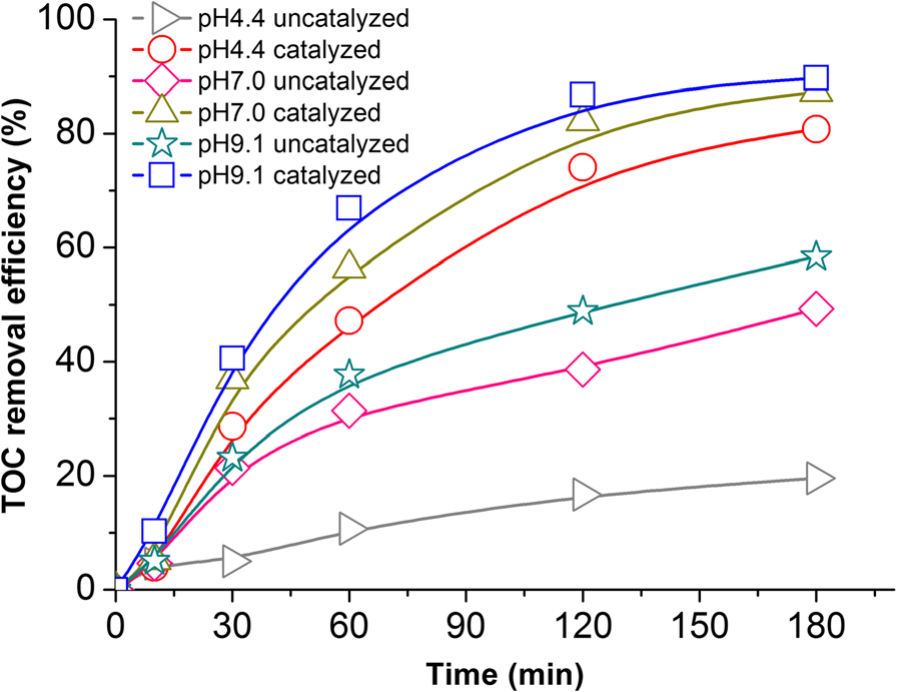
**Influence of pH on TOC removal during catalyzed and uncatalyzed alachlor ozonation: (▹) pH 4.4 uncatalyzed; (○) pH 4.4 catalyzed; (◇) pH 7.0 uncatalyzed; (∆) pH 7.0 catalyzed; (☆) pH 9.1 uncatalyzed; (□) pH 9.1 catalyzed.** T: 20°C, Alachlor concentration: 100 mg L^-1^.

As mentioned above, pH dramatically affected the ozonation of alachlor. Therefore, the effects of the initial pH of the reaction solution on the uncatalyzed and Cu/Al_2_O_3_-honeycomb catalyzed ozonation were investigated. As shown in Figure 6, the enhancing effect on TOC removal was observed in two ozonation processes with the initial pH of the solution increased. This enhancing effect resulted from the increased amount of decomposing ozone and the corresponding larger output of •OH at higher pH levels. Even though a large amount of •OH was produced in the Cu/Al_2_O_3_-honeycomb catalyzed ozonation, the enhancing effect of pH was still obvious.

Higher TOC removal efficiency was obtained in Cu/Al_2_O_3_-honeycomb catalyzed ozonation than uncatalyzed ozonation (the TOC removal percentage was increased from 20.0% to 90.1%, Figure 6). Even at pH 4.4 in the catalytic process, a higher alachlor mineralization percentage was achieved than at pH 9.1 in the uncatalytic process. This result demonstrated that Cu/Al_2_O_3_-honeycomb has high catalytic efficiency in the ozonation of alachlor. It was also verified that the •OH produced in Cu/Al_2_O_3_-honeycomb catalyzed ozonation contributes much more to alachlor mineralization than does the decomposition of ozone in the uncatalyzed one. Furthermore, we used ICP-AES to measure the concentration of Cu, and the detection limit is 2 g L^-1^ at 324.75 nm. Only about 2 g L^-1^ Cu^2+^ releasing from the Cu/Al_2_O_3_ catalyst was detected in solution after being used for 3 times, and less metal loss was observed with the next continuous application of the catalyst. When the operations were repeated by 10 times, there was no Cu^2+^ detected in water. The total leaching amount is 2 g L^-1^, which is about 1.8% of the loading amount on the support. It could be deduced that the coated Cu/Al_2_O_3_ catalyst was nearly stable and applicable.

## Conclusions

This study has shown that Cu/Al_2_O_3_-honeycomb is a feasible and efficient catalyst in the ozonation of alachlor in water. The advantage of Cu/Al_2_O_3_-honeycomb was easily solid–liquid separation with powered Cu/Al_2_O_3_ didn’t have. Solid, liquid and gas phases contacted sufficiently through pores of Cu/Al_2_O_3_-honeycomb in the experiment of Cu/Al_2_O_3_-honeycomb. Three-phase contacted with a bottom-up sequence, more catalyst can react with alachlor. However, powdered Cu/Al_2_O_3_ catalyst presented suspended state without rules in the solution with the impact of air. Part of the catalyst can’t suspend so that the treatment effect is not ideal. The catalyst was capable of increasing the removal percentage of TOC from 20.0% in uncatalyzed ozonation to 75.0% in a catalyzed ozonation process in the same pH conditions. Less intermediate oxidation product (aliphatic carboxylic acids) was produced in the catalytic process than in the uncatalytic one, leading to higher mineralization efficiency. Increasing pH of the reaction solution could enhance the catalytic efficiency of Cu/Al_2_O_3_-honeycomb on the ozonation of alachlor.

## Abbreviations

MCL: Minimum contamination level; TOC: Total organic carbon; XRD: X-ray diffractometry; IC: Ion chromatograph; ACN: Acctonitrile; DMPO: Dimethyl pyridine N-oxide; EPR: Electron paramagnetic resonance.

## Competing interests

The authors declare that they have no competing interests.

## Authors’ contributions

HL formulated the research, planned the experiment and carried out the degradation of alachlor in the uncatalyzed ozonation process. YH carried out the catalyzed ozonation process of alachlor and chemical analysis. SC carried out the variation of pH in alachlor ozonation process and the effect of pH on ozonation and prepared figures and tables. All authors participated in the article’s design and coordination and helped to draft the manuscript. All authors read and approved the final manuscript.
